# Categorizing datasets of road traffic accidents in Oman spanning from 2012 to 2022

**DOI:** 10.1016/j.dib.2024.110184

**Published:** 2024-02-13

**Authors:** Hussin A. M Yahia, Ali Ahmed Mohammed, Taleb Eissa, Shaban Ismael Albrka, Mohd Azizul Ladin, Hisham Jashami

**Affiliations:** a Department of Civil & Mechanical Engineering, Middle East College, Muscat, Oman; b Department of Civil Engineering, Lassonde School of Engineering, York University, Toronto, Canada; c Department of Civil Engineering, Florida Institute of Technology Melbourne, Florida, USA; d Department of Civil Engineering Near East University Lefkoşa, North Cyprus; e Faculty of Engineering UMS, University Malaysia Sabah, Kota Kinabalu, Malaysia; f School of Civil and Construction Engineering, Oregon State University, 101 Kearney Hall, Corvallis, OR 97331, USA

**Keywords:** Number of accidents, Number of injuries, Number of deaths and traffic crash statistics

## Abstract

Road traffic accidents constitute the primary cause of fatalities associated with injuries and engender substantial economic ramifications for affected individuals, their families, and entire nations. The Sultanate of Oman, like other countries, suffers from traffic accident injuries and traffic congestion. The accident rate for the period 2021 was recorded as one accident every six hours. Despite a 70% increase in total number of vehicles and an 81% rise in licensed drivers between 2012 and 2019, data on traffic accidents demonstrate an improving trend with a notable 55% decline in crash fatalities. However, it is important to recognize that road traffic accidents in Oman encompass not only social issues but also pose a significant economic burden, resulting in substantial financial costs for the nation. Notwithstanding, it was discovered that more than 50% of fatal crashes in Oman were primarily caused by excessive speeding. The main goal of this research is to analysis the causes and trends of traffic accidents at the national level in the Sultanate of Oman. Data analysis reveals speed as the primary cause of traffic accidents in Oman, with Muscat and Dhofar registering the highest accident rates. In addition, the distribution of deaths and injuries resulting from accidents varies according to Gender and nationality. According to the road accident scenario analysis in the state, more traffic accidents occurred in males than females. Traffic accidents have witnessed a notable decline over the past decade, attributable to the diligent efforts and interventions implemented by the Royal Oman Police.

Specifications Table

**Table:** 

Subject	Civil Engineering, Transportation Engineering
Specific subject area	Road Traffic Accidents, traffic safety
Data format	Raw, Analyzed
Type of data	Table,.csv file.
Data collection	Data was collected from the National Center for Statistics and Information (NCSI) in Oman and distributed along with the Sultanate of Oman cities
Data source location	• Country: Sultanate of Oman • Coordinates: 21° 00′ N latitude and 57° 00′ E longitude
Data accessibility	The data are available in this article. Repository name: Mendeley Data identification number: DOI: 10.17632/zzw599rty5.1 Direct URL to data: Omani Road Traffic Accident Dataset Spanning From (2012 –2022) - Mendeley Data

## 1 Value of the Data


 

• Road engineers or researchers could use the data as a useful tool for prospectus research.
• This information may serve as a catalyst for researchers to share their statistics to enrich the information needed for the research in the Sultanate of Oman.
• A thorough statistical analysis could be performed to get more useful data.
• The information may be useful in reducing the Sultanate of Oman's rising injury rate in the Sultanate of Oman.
• Data can be helpful in predicting the number of road accidents in Oman in the future.
• Policymakers can use the data to gain a better understanding of the evolution of traffic accidents in Oman, which could be helpful for further study.

## 2 Background

Roughly 22 percent of deaths globally are attributed to traffic accidents, which are ranked as the ninth most common cause of mortality. In traffic accidents, approximately 13.1 million people lose their lives annually, or 3287 people every day on average. In the world, Oman is ranked 57th in terms of the frequency of road accidents, injuries, and deaths, while ranking fourth among the states of the Arab Gulf Cooperation Council (GCC). Of the Gulf states, Oman has the highest rate of traffic accidents. As road accidents continue to be a leading cause of death and injury worldwide, there is an urgent need for comprehensive data to better understand the patterns and underlying factors contributing to these accidents in Oman. Data on road accidents, including accidents, deaths, and injuries, were collected by the National Center for Statistics and Information (NCSI) and the Royal Oman Police (ROP).

The data were classified into number of traffic accidents, injuries and deaths by province and type, number of traffic accidents, injuries and deaths by province and nationality and number of traffic accidents according to the years proposed in this study from 2012 to 20,122. Injuries and deaths by cause. The goal of compiling datasets is to help advance traffic safety efforts by creating a reliable and accessible resource for stakeholders, researchers, and the public to use in efforts to mitigate the devastating effects of traffic accidents.

## 3 Data Description

The exploration of oil and gas in Oman has brought about a transformation in the economy, resulting in a notable rise in private vehicle ownership. This trend is closely associated with the rapid growth of drivers and vehicles, paralleling the substantial increase in the country's Gross Domestic Product (GDP) and per capita income [1]. The data on traffic accidents, including accidents, deaths, and injuries, as well as the number of cars in each city within the Sultanate of Oman, were collected from the National Center for Statistics and Information (NCSI) and the Royal Oman Police (ROP). Compared to 2019, when private vehicles accounted for approximately 79.4% of the total vehicle count with 1553 vehicles on the roads, there was a 3.4% increase, resulting in private vehicles representing about 77.8% of all vehicles in the Sultanate of Oman 1547,741 vehicle. By January of the most recent year 2022, the number of vehicles on the roads had reached 1603,376, reflecting a 55,635,000 vehicle increase.

**Fig. 1:**
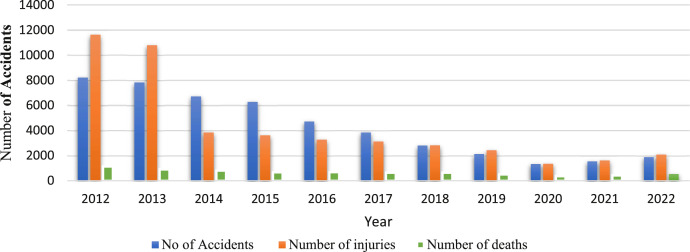
Traffic accidents were distributed throughout the study period.

However, the statistics of the National Center for Statistics and Information (NCSI) indicated a decrease in the number of injuries, accidents, and deaths in 2022 compared to 2012 as shown in Fig. 1. However, in Muscat, the rate of accidents, injuries, and deaths showed a significant increase compared to other cities in Oman. In 2018, out of the total 2802 accidents recorded in Oman, 797 accidents occurred in Muscat, accounting for a rate of 28.4%. This surge can be attributed to factors such as high population density, a large number of vehicles, and the concentration of expatriates and residents in the capital city of Muscat. Speeding, reckless behaviour, and negligence emerged as predominant factors contributing to traffic accidents in Muscat, as evidenced by a total of 4721 recorded accidents in 2016. Notably, 2499 of these accidents were directly attributed to speeding.

The statistics reflect the efficacy of the Royal Oman Police's efforts and recommendations aimed at enhancing driver behaviour, reducing road traffic accidents, and providing comprehensive services and instructions to aid all road users in preventing accidents. Furthermore, citizens and residents have shown an increasing sense of responsibility in mitigating the risks associated with traffic accidents, aligning with the Royal Oman Police's endeavors to minimize such incidents and their consequential harm. Notably, the youth category appears to be the most impacted by these accidents [2].

The increase in the number of vehicles on the road can be attributed to population growth, economic development, and social activity. Consequently, this rise has become one of the primary factors contributing to the upsurge in traffic accidents. Additionally, the growing population of expatriates from diverse cultural backgrounds has also played a role in this trend. Oman, situated on the southern shore of the Asian Arab Peninsula, shares borders with Yemen to the southwest, the United Arab Emirates to the northwest, and Saudi Arabia to the west. Furthermore, it offers scenic views of the Oman Sea and the Arabian Sea to the northeast and southeast, respectively. The population of Oman, consisting of both Omanis and expatriates, stands at approximately 4933,850 million (Statistics 2022).

**Fig. 2:**
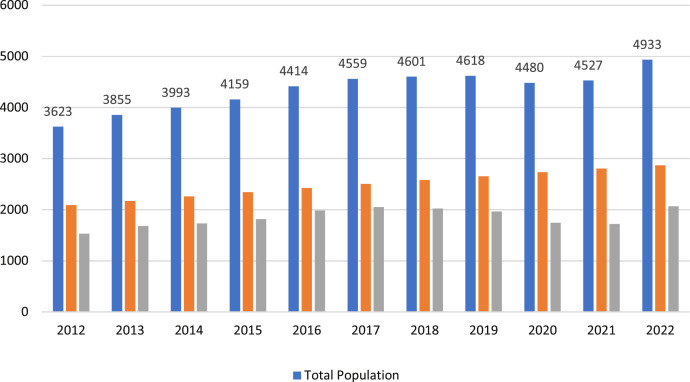
Total of population (Omani and expatriates).

**Fig. 3:**
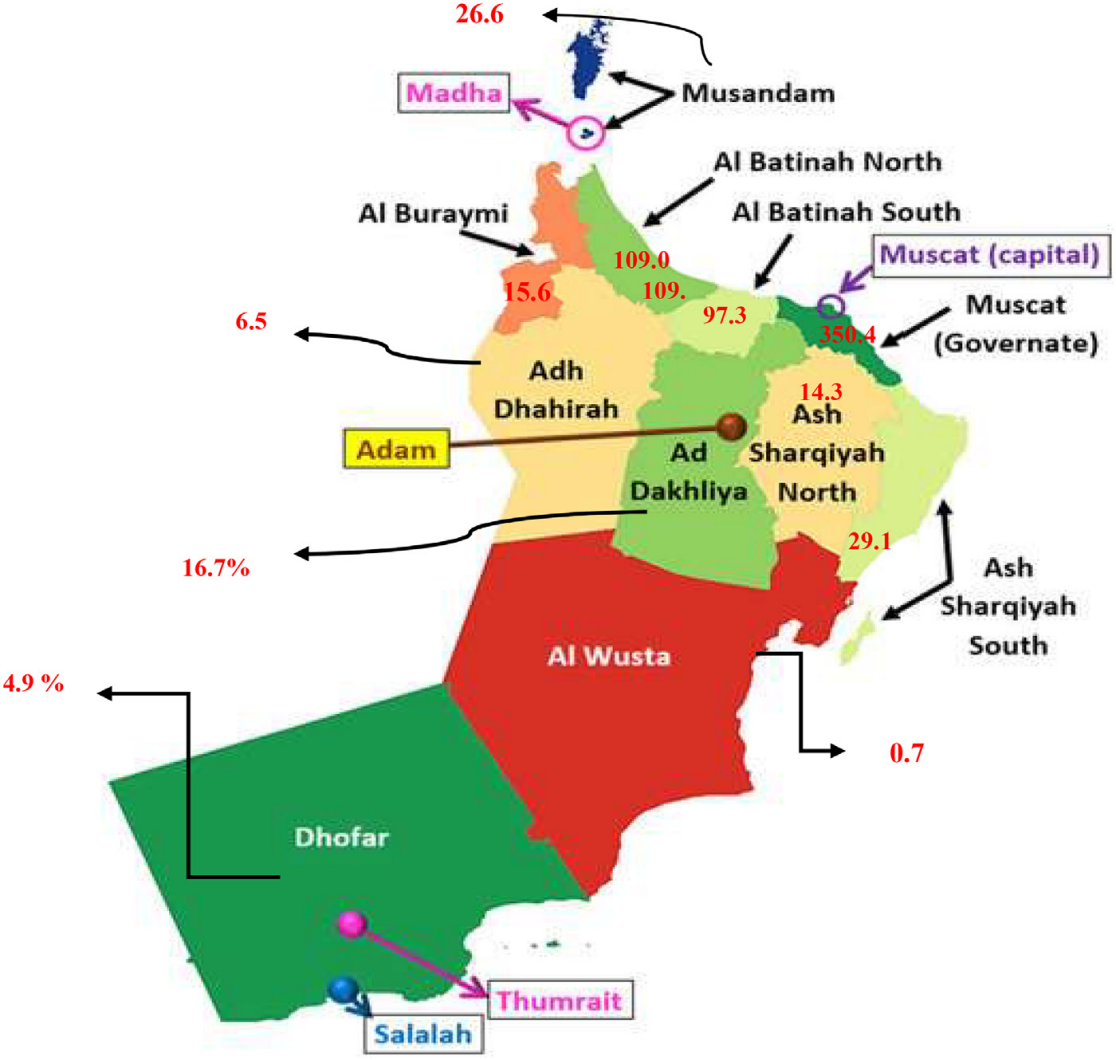
Population density by governorates (Persons/km^2^), 2022.

Oman's population density increased to 14.15 people per km^2^ in 2020 CE, and the Governorate of Muscat continued to lead the Sultanate's governorates in terms of population, with a total population of 4933,000 people, up from (3623,000) in the 2012 census and an increased rate of (73%) as shown in Fig. 2. 7.6% of people reside in urban areas, compared to 13.4% who reside in rural areas. Of the country's population, 34.0% is female and 66.0% is male. The administrative division of the Sultanate of Oman, which is one of the distinctive features of the modern state, has been approved into 11 governorates, as shown in Fig. 3.

In this research, data mining techniques were applied to classify the severity of traffic accidents on Omani roads. The traffic accident data includes traffic accidents involving drivers, pedestrians, and passengers between 2012 and 2022 in all governorates in Oman. Road accidents in Oman are divided into three categories (number of accidents, number of injured and number of deaths). The dataset was classified by number of traffic accidents, injuries and deaths by province and type (drivers, pedestrians, and passengers), number of traffic accidents, injuries and deaths by province and nationality (Omani or expatriate), and number of traffic accidents, injuries, and deaths by causes. The data files (read in Excel format) were presented in Tables 1 and 2, respectively, and deposited with Mendeley Data. Table 3 contains the number of traffic accidents, injuries, and deaths by cause. It is noted that the number of road accident accidents was higher than other types of accidents. However, the total number of fatal accidents was the lowest among all other types, which makes sense.

**Table 1:** Summary statistics of the dataset.

	Number of accidents	Number of injuries	Number of deaths
Number of Parameters	11	11	11
Minimum (#)	1341	1365	371
Maximum (#)	8209	11,618	1139
Sum (#)	47,269	46,597	7360
Mean (#)	4297.18	4236.09	669.09

**Table 2:** 95% confidence interval for the mean.

Type	Number of samples	Mean	Standard Deviation	95% C.I
Number of accidents	11	4297.18	2589.446	846, 5827
Number of injuries	11	4236.09	3539.400	1660, 7162
Number of Deaths	11	699.09	211.864	−3465, 1847

**Table 3:** Analysis of variance (ANOVA) table.

Source of variation (SV)	Degree of freedom (df)	The sum of square (SS)	Mean square (MS)	F	*P*-value
Types	2	340,472.87	2,670,236.44	7.43	0.003
Error	18	16,117,296.06	1,343,108.005		
Total	20	16,457,769.93			

### 3.1 Materials and methods

**Fig. 4:**
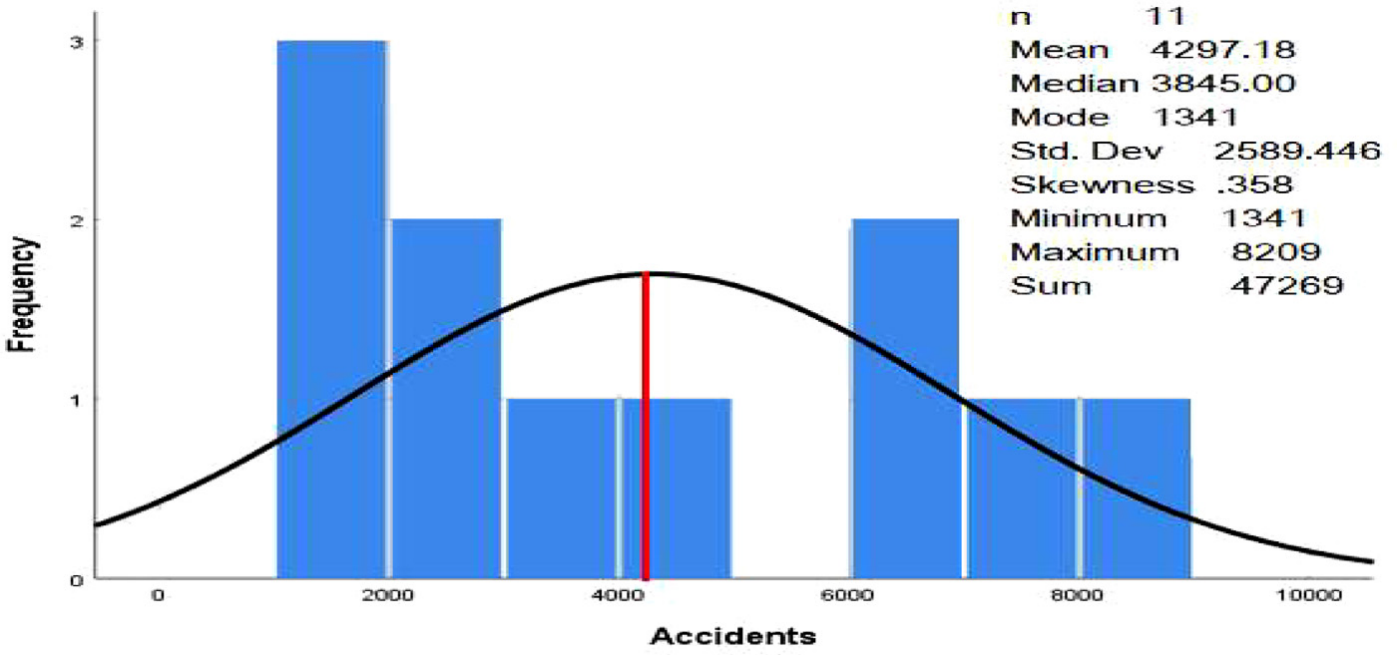
The histogram for number of accidents.

**Fig. 5:**
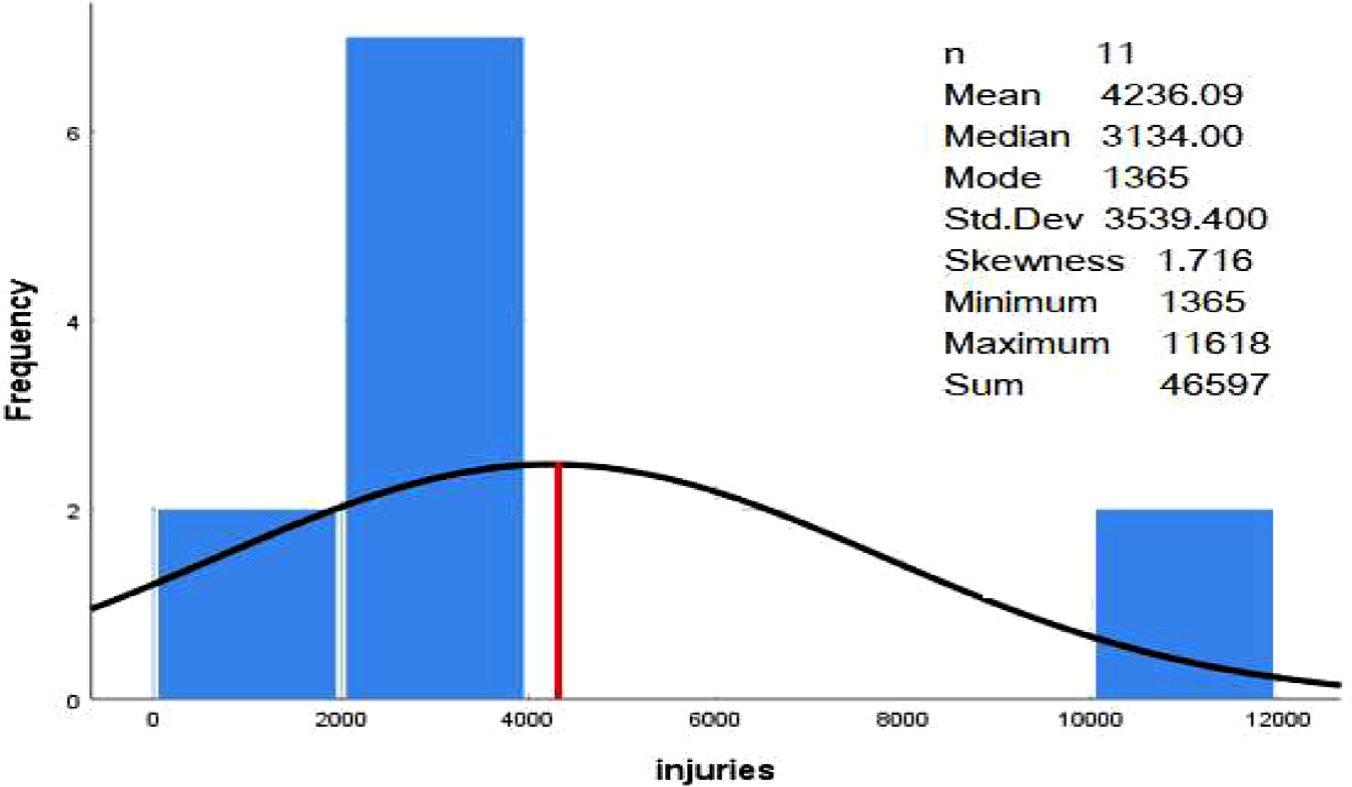
The histogram for the number of injuries.

In this study, descriptive statistics were used to summarize the data and create graphs to facilitate understanding and summary. SPSS version 25 was used for data analysis. In order to confirm the mean influences of the different types of accidents, a one-factorial ANOVA is carried out in this study [3,4].

(1) $$Y_{IJ}=\mu +\alpha_{IJ}+e_{ij}$$

where *Y_ij_* is the *j*th observation in the *i*th treatment, μ is the overall mean, αij is the effect of treatment *i, e_ij_* is the error term [4] However, Minitab version 20 was used for the analysis of variance (ANOVA), and further tests (Fig. 4, Fig. 5).

Variance analysis (ANOVA) and additional tests were conducted using Minitab version 20. Additionally, all analyses used a significance level of 0.05. Table 3 shows the outcome.

### 3.2 Traffic accident datasets

The number of traffic accidents, the number of injuries and the number of deaths were organized into spreadsheets, including the following.

1. Number of Traffic Accidents, Injuries, and Deaths by Governorates & Type
2. Number of Traffic Accidents, Injuries, and Deaths by Governorates and Nationality
3. Number of Traffic Accidents, Injuries, and Deaths by Causes.

Spreadsheet showcasing the statistical distribution of accidents categorized by their nature of occurrence.

**Fig. 6:**
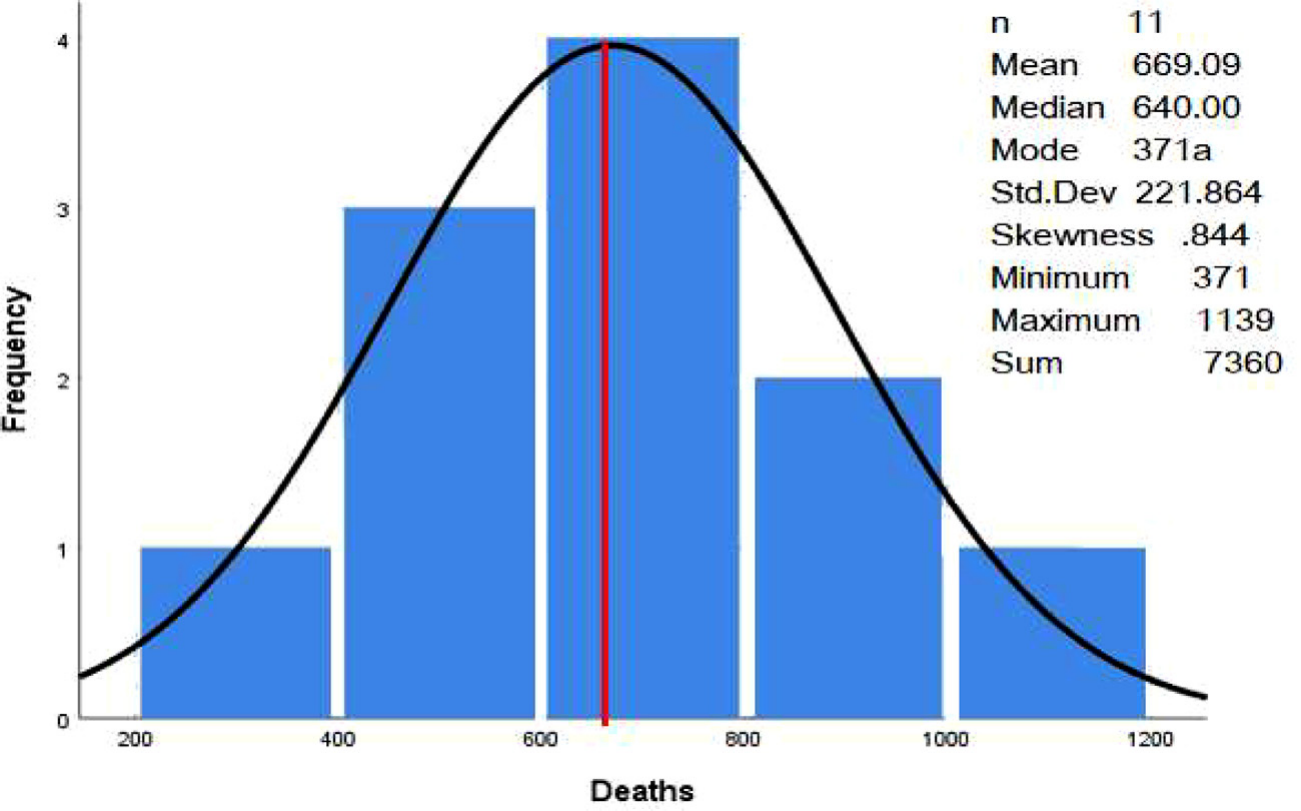
The histogram for the number of deaths.

Fig. 6 clarified the distribution of traffic accidents, injuries, and deaths by the governorate in the Sultanate of Oman, Muscat recorded the highest rate of traffic accidents with 2759 traffic accidents (33.60%) in 2012. This may be attributed to the high number of vehicles on the roads in the capital, Muscat, as well as the population density compared to other Omani cities. It was observed that there is a slight increase in the percentage of accident types of collisions between vehicles and fixed collision objects. Al Batinah North and Muscat recorded the highest rate of 220 deaths at (19.31%) and Muscat 157 deaths at (13.78%) in 2012, then deaths recorded a gradual decline in the year 2022, as shown in Fig. 6.

**Distribution of traffic accidents across governorates, categorized by nationality.**

**Fig. 7:**
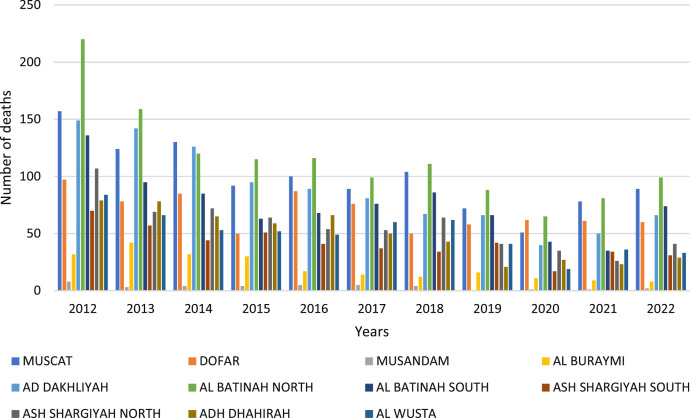
No. of traffic accidents deaths by governorates.

**Fig. 8:**
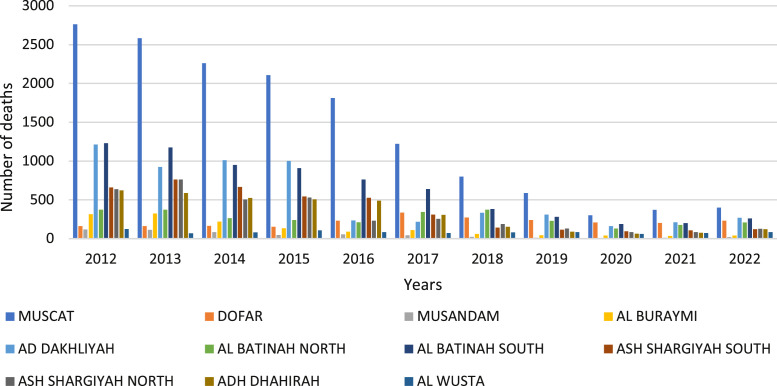
No. of traffic accidents by governorates.

**Fig. 9:**
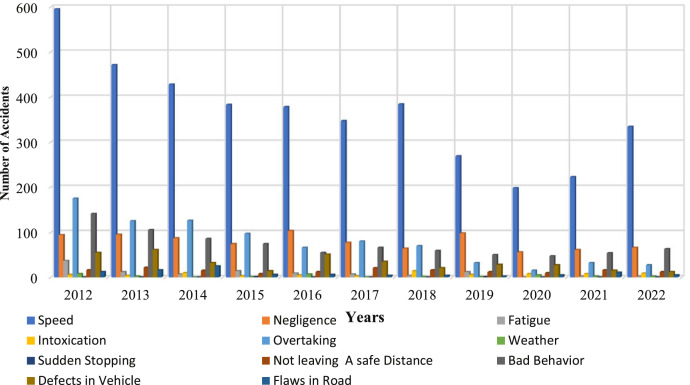
No. of traffic accidents by causes.

Death accidents for expatriates on the roads by governorate and nationality in Oman recorded an increase in the governorates of North Al Batinah (15.74%) and Muscat (19.42%) in 2012 as shown in Table 4. It was noted that men died at a higher rate than women. More males than women lose their lives in automobile accidents each year. Males are more prone than females to drive longer distances and participate in risky driving practices, like speeding and not using seat belts (Fig. 7, Fig. 8, Fig. 9).

**Table 4:** Number of deaths by nationality 2012.

Governorates	Number of deaths					
	Expatriate			Omani		
	Total	Female	Male	Total	Female	Male
MUSCAT	74	5	69	83	18	65
DOFAR	46	10	36	51	5	46
MUSANDAM	3	0	3	5	0	5
AL BURAYMI	17	2	15	15	2	13
AD DAKHLIYAH	32	1	31	117	14	103
AL BATINAH NORTH	60	9	51	160	33	127
AL BATINAH SOUTH	42	4	38	94	9	85
ASH SHARGIYAH SOUTH	16	3	13	54	10	44
ASH SHARGIYAH NORTH	33	1	32	74	15	59
ADH DHAHIRAH	17	4	13	62	15	47
AL WUSTA	41	6	35	43	1	42
TOTAL	381	45	336	758	122	636

**No. of Traffic Accidents, Injuries, and Deaths by Causes**

In Oman, traffic accidents are a serious problem, and the main causes of these mishaps are speeding and inappropriate attitude. According to recent data Oman's death rate has been rising, necessitating increased awareness and preventative measures. In Oman, speeding plays a big role in a lot of traffic incidents. Drivers who exceed the speed limit endanger not just other people but also themselves in collisions that can cause serious injuries or even fatalities. On Omani roads, there are also a lot of accidents caused by aggressive and careless driving practices like tailgating, weaving in and out of traffic, and ignoring traffic signals. In Oman, the number of people killed and injured in road accidents has alarmingly increased in the last few years. Even while measures to raise awareness and enforce traffic regulations have been implemented to improve road safety, much more needs to be done to address the underlying causes of these accidents. To reduce accidents and safeguard themselves and other people on the road, drivers must put safety first and follow traffic laws. Additionally, spending money on infrastructure upgrades and enforcing harsher traffic-related penalties can help to lessen accidents and save lives. In conclusion, the most frequent reasons for traffic accidents in Oman and some developing countries that result in injuries and fatalities are speeding and bad behavior [5]. Many variables have been found to be significant and intuitive, related to roadway geometry, traffic control, pavement characteristics, temporal attributes, crash-specific characteristics, and environmental conditions. To encourage responsible driving behavior and foster a culture of responsibility on the roads, authorities and the general public must collaborate. As seen in Fig. 6, Oman can significantly advance road safety and lessen the number of traffic accidents by addressing these problems.

## Limitations

Not applicable.

## Ethics Statement

The work described above didn't involve human or animal subjects; Therefore, no regulatory compliance guidelines were applicable.

## CRediT authorship contribution statement

**Hussin A. M Yahia:** Investigation, Data curation, Writing – original draft. **Ali Ahmed Mohammed:** Investigation, Data curation, Writing – review & editing. **Taleb Eissa:** Investigation, Writing – review & editing. **Shaban Ismael Albrka:** Investigation, Writing – review & editing. **Mohd Azizul Ladin:** Investigation. **Hisham Jashami:** Investigation, Writing – review & editing.

## Data Availability

No. of Traffic Accidents- 2012-2022 (Original data)
